# Supplementary material for: Patterns of Intron Gain and Loss in Fungi
Source: PLoS Biol. 2004 Nov 30;2(12):e422. doi: 10.1371/journal.pbio.0020422 (PMC532390; doi:10.1371/journal.pbio.0020422)
Supplement: Table S1 — Also available at http://genes.mit.edu/NielsenEtAl/. (4.3 MB ZIP). [file pbio.0020422.st001.zip › NielsenEtAl/html/861.html]

AN2468.1.NCU04435.1.MG05107.1.FG02767.1


```
 CLUSTAL W (1.82) Multiple Sequence Alignments - Introns Inserted


Sequence 1: NCU04435.1	536 aa
Sequence 2: MG05107.1	547 aa
Sequence 3: FG02767.1	652 aa
Sequence 4: AN2468.1	508 aa
Alignment Length: 683 aa
Number Identitical Residues: 159 aa
Alignment Score (without introns) 10701


MG05107.1 	MSVRPRPEGLLEPRRPGGNGEYRKTTDAEDVTTAEPLSDGGYRDFS~DIDPDSGVKRGLK
NCU04435.1	MSLSQK----------------GSATILDDHETAIPSKDDG-GDFR~AIDPHSGVKRGLK
FG02767.1 	MAFPHNKD---------DDVVAGDGPSAYDNYDSTRLNDTS--EFG~SIDPDSGVKRGLK
AN2468.1  	MALWKD----------------QTSQIQNEVDEASPGSNHDYDHDP2ALDEQE--ERGVA
          	*:.                          :   :   .: .  .    :* ..  :**: 

MG05107.1 	TRHLSMMALAGIIGPGLLIGSGGALASGGPAALIIGFGVIG~IVAFSIMQSLGELTTLYP
NCU04435.1	TRHLSMMALAGIIGPGLLVGSGGALHSGGPASLLIGFGVIG~LIAFSIMQSLGELTTLYP
FG02767.1 	NRHLSMMALAGIIGPGLLVGAGGALNVGGPAALLIGFGVVG1IIAFCIMQSLGEVTTLYP
AN2468.1  	KCHR-------------------------------------~-------QALGEMATAIP
          	. *                                              *:***::*  *

MG05107.1 	TGGAFTSLSSRFVDKAFGVAVGWNYYII~WWAVLSNEYNVICSIFNLWGSQVPIWGYFLI
NCU04435.1	SGGAFTDLGDRFIDKAFGVAVGWNYLII1WVCVLANEYNVISSVMVDWSDKVPLWGYFLI
FG02767.1 	GGGSFISLAERMVDKSFSVAVGWNYFVI~WAAVLANEYNVICSILTYWGPVVPLWGYFLI
AN2468.1  	VTGSFTEYAQRFVDDSLAFGLGWAYWYL~WVTVLANEYNAVSLVIMYWTDAVPQWAWILI
          	  *:* . ..*::*.::...:** *  : *  **:****.:. ::  *   ** *.::**

MG05107.1 	FW0-------LLGVESFGEAEFWLAL------------------LKLIGLVAYFIFSLV~
NCU04435.1	FW0FAFLGFQLLGVETFGEAEFWLAL------------------IKLMGLVAYFIFSIV2
FG02767.1 	FW~TVFMAFQLLGVEAFGEAEFWLAL------------------MKLLGLTAYFIFAII~
AN2468.1  	FW~ILFLGLANFGVRAYGEMEFWLSLYVSPCVRLVKRALADPRRIKVLALIVFFILAIC~
          	**     .   :**.::** ****:*  :..    . : :..  :*::.* .:**:::  

MG05107.1 	YVGGGIPG-APALGSRYWHDPGAF-ANGFKGVATVFVFCSTFYAGVES~VAVAATETKNP
NCU04435.1	YVSGGVKG-ADAIGFKYWRDPGPF-ADGFRGVASVFVFCSTFYAGVES~VAVAATETKNP
FG02767.1 	YAAGGLVGQDEPLGFRYWSDPGAFNGNGFRGVAAVFVFCATFYSGVES~IAVAATETRNP
AN2468.1  	ISTGGIGG--DVIGFKYWHNPGAF-ADSINGVARTFVVAGTLYAGTEM2VGITAGESSNP
          	   **: *    :* :** :**.* .:.:.*** .**...*:*:*.*  :.::* *: **

MG05107.1 	RTAIPLAIRQVFWRIVFVYMG~SAIFFGLTCPWDADGLVSGENRALRSPMTVAIQNAGWQ
NCU04435.1	GVAVPHSIRQVFIRIVVVYMG~CAFFFGLTCPSNAPDLSGGASHALKSPMTIALHNAGWA
FG02767.1 	GVAVPQAIRQVFWRIIFVYMG~SALFFGITCPANASGLVNGGSKALQSPMTIAIENAGWQ
AN2468.1  	QKAVPIAIRQVFWRILVFYIG1TMFFIGILMPYTEPQLLNSSSYGANSPLTIALSSAGIL
          	  *:* :***** **:..*:*   :*:*:  *     * .. . . .**:*:*: .**  

MG05107.1 	SG~VHLINAFIFVTCLSAINSSIYIASRTILFMAQSQMAPKFLGWTDRRGVPIPAILFAN
NCU04435.1	NG~THLINAFIFVTCLSAVNSSIYIGSRTLLYMAQDGKAPRFLGWTDRRGVPVYAIIFTN
FG02767.1 	GG1VHLINAFILLTCLSAINSSIYFGSRTVFYMAQSGKAPKIFAWTNKRGVPVWAILITN
AN2468.1  	PA~AHLINALIVVSVISAGIGSLYVASRTILFMARNGKAPKILGRTNAAGVPWVAIIFSN
          	 . .*****:*.:: :**  .*:*..***:::**:.  **:::. *:  ***  **:::*

MG05107.1 	ACGSLSMMNISTGAATAYSYIVNLSGVSTFLVWGSISFTHIRFRTAWQAQGFDPQSLPFR
NCU04435.1	ACGALSMMNVSTGASKAYGYIINLSGVSTFLVWGAISLIHIRFRAAWKAQGHSPEELPYK
FG02767.1 	AVGAISMMNVSTGASKAYSYIVNLSGVSAFLVWGSICFIHIRFRRAWVAQGRSLDELPYK
AN2468.1  	IFTCIVFLTLGESAGKVYNALITLSGVATFLVWTSICIAHIRFRAALTAQGQSPSTLPFR
          	   .: ::.:. .*...*. ::.****::**** :*.: ***** *  *** . . **::

MG05107.1 	SMLYPWNAYFGLGANVFLALVQGWST-LAPFDAPTFVDAYILIPLFAVIYVAYKFAFKTR
NCU04435.1	SLWYPWNAYFGLISNVFLAFVQGWST-LAPFDAGNFVDAYILLPLFPVIYFVYKFTFKTR
FG02767.1 	ALGFPYLAWFGLAACIFLALVQGWTT-LSPFDAGNFVDAYILVPLFGIIYVFCKILWRGS
AN2468.1  	AALYPYGTYFALVLNIFMIFFQGYTAFLNPFSSDDFVINYILLPVFVLFVVGWKVWHKTK
          	:  :*: ::*.*   :*: :.**::: * **.:  **  ***:*:* :: .  *.  :  

MG05107.1 	--FWRAEEIDLLSGRRRDLEEDREIQNRTPSGAPLSAWQR~-----------------LW
NCU04435.1	--LWRPHEVDLQAGRRRDLDEAKEVE-RGADGRPLPWWRK~-----------------IF
FG02767.1 	DPLKRSWSIDLDSGRRTDLDQKSGPTSRDLPG-QMPWWRK0RNDGDKLSRYRTESHSHLF
AN2468.1  	--IVRLEDMDIWTGRRERAPEVEESSRWKGKGWREKVWRG~-----------------VV
          	  : *  .:*: :***    :          *     *:                   : 

MG05107.1 	KNF------------------------------------------------~-----~--
NCU04435.1	SSL------------------------------------------------~-----~--
FG02767.1 	PFYHRNLGNFTAGILGRSLESRLCDGNSVWMPAAAIWRHSVGDVRWAVLKG2LYCER2IC
AN2468.1  	G--------------------------------------------------~-----~--
          	                                                            

MG05107.1 	------~--~--------------------------
NCU04435.1	------~--~--------------------------
FG02767.1 	CDFDGY2ID1VFQSTEIALISVPFFYYSKQRTTTNR
AN2468.1  	------~--~--------------------------
          	
```
